# Fabrication of Transgelosomes for Enhancing the Ocular Delivery of Acetazolamide: Statistical Optimization, In Vitro Characterization, and In Vivo Study

**DOI:** 10.3390/pharmaceutics12050465

**Published:** 2020-05-20

**Authors:** Eman A. Mazyed, Abdelaziz E. Abdelaziz

**Affiliations:** Department of Pharmaceutical Technology, Faculty of Pharmacy, Kaferelsheikh University, Kaferelsheikh POB 33516, Egypt; Abdelaziz_abdrabo@pharm.kfs.edu.eg

**Keywords:** ACZ, transfersomes, transgelosomes, corneal drug delivery

## Abstract

Acetazolamide (ACZ) is a potent carbonic anhydrase inhibitor that is used for the treatment of glaucoma. Its oral administration causes various undesirable side effects. This study aimed to formulate transgelosomes (TGS) for enhancing the ocular delivery of ACZ. ACZ-loaded transfersomes were formulated by the ethanol injection method, using phosphatidylcholine (PC) and different edge activators, including Tween 80, Span 60, and Cremophor RH 40. The effects of the ratio of lipid to surfactant and type of surfactant on % drug released after 8 h (Q_8h_) and entrapment efficiency (EE%) were investigated by using Design-Expert software. The optimized formula was formulated as TGS, using poloxamers as gelling agents. In vitro and in vivo characterization of ACZ-loaded TGS was performed. According to optimization study, F8 had the highest desirability value and was chosen as the optimized formula for preparing TGS. F8 appeared as spherical elastic nanovesicles with Q_8h_ of 93.01 ± 3.76% and EE% of 84.44 ± 2.82. Compared to a free drug, TGS exhibited more prolonged drug release of 71.28 ± 0.46% after 8 h, higher ex vivo permeation of 66.82 ± 1.11% after 8 h and a significant lowering of intraocular pressure (IOP) for 24 h. Therefore, TGS provided a promising technique for improving the corneal delivery of ACZ.

## 1. Introduction

Glaucoma is considered the second most common cause of blindness after cataract. Glaucoma is usually associated with high intraocular pressure (IOP), which leads to damage to the optic nerve [1]. Acetazolamide (ACZ) is used orally in large doses for the treatment of glaucoma, by decreasing the IOP. The mechanism of action of ACZ involves inactivation of carbonic anhydrase and interference with the sodium pump that lowers the aqueous humor formation and hence decreases IOP [2]. However, it has undesirable side effects, such as renal failure, metabolic acidosis, and central nervous system depression. Many researchers such as Nagasubramanian et al. [3] and Kaur et al [4] reported that the topical delivery of ACZ could decrease the IOP without any systemic side effects due to its local effect in the eye. Therefore, the topical delivery of ACZ is more acceptable than oral delivery. Unfortunately, ACZ is practically insoluble in the aqueous lachrymal fluid, which hinders its topical delivery [5].

Several attempts have been adopted to improve the topical delivery of ACZ, such as using contact lenses [6], surfactant gel [7], cyclodextrins [8], viscolyzers [9], penetration enhancers, bioadhesive polymers, and cosolvents [10]. These drug-delivery systems provide various advantages over conventional drug therapy, yet they suffer from different problems, including the difficulty of administration, low patient compliance, toxicological complications, poor corneal penetration, and tissue irritation [11].

In order to overcome these pitfalls, many researchers have recommended the formulation of vesicular/nano drug-delivery systems for improving the ocular delivery of ACZ. Liposomes are considered to be an effective nanovesicular system that could improve the low corneal permeation and poor bioavailability of ACZ. Liposomes offer several potential advantages over other ophthalmic delivery systems, including biodegradability, safety, and non-toxicity. Moreover, liposomes could provide prolonged drug release, increasing the contact time with corneal and conjunctival surfaces, and they could protect the drug molecules from degradation by the metabolic enzymes in the eye [5].

Transfersomes are ultra-flexible deformable nano-liposomes. They are biodegradable and biocompatible nanocarriers because they are mainly made from natural phospholipids. In addition, they have high encapsulation efficiency and could protect the entrapped drug from metabolic degradation [12]. Transfersomes are more advantageous than conventional liposomes because they are obtained by adding surfactants to the phospholipid bilayers of conventional liposomes. The edge activators act as destabilizing factors in the vesicular membrane that increases the flexibility and deformability of the transfersomal vesicles, allowing them to squeeze through small pores of the skin layers without rupture [13] and consequently increase drug permeability and bioavailability [12,14].

Ophthalmic solutions are disadvantageous because of their rapid drainage. Therefore, these solutions could not maintain an adequate drug concentration in the precorneal area [15]. In situ gel-forming solutions are phase-transition systems that are administered in a liquid form and converted to a gel phase in the eye. Gel-forming solutions can improve the precorneal residence time of a drug and thus realize higher bioavailability [16]. Poloxamer 407 (Pluronic^®^ F127) is a block copolymer that consists of polyethylene oxide (PEO) and polypropylene oxide (PPO) units. It is known to exhibit reverse thermal gelation below a certain concentration and temperature. At a concentration of 18% (w/w) or higher in aqueous solution, poloxamer 407 is transformed from a low-viscosity solution to a gel at ambient temperature. Poloxamer 188 (Pluronic^®^ F68) is considered to be a regulatory substance that helped increase the gelling temperature (GT) of poloxamer 407 [17].

Since prolonged residence is required for better activity, various researches have been made for using hydrogels as a vehicle for the incorporation of nanoparticles, such as lipogelosomes that are formulated by dispersion of liposomes in a suitable medium containing gelling agents. Lipogelosomes are advantageous over conventional liposome formulations because they provide a double encapsulation strategy, leading to more controlled drug release. In addition, lipogelosomes improve the stability of liposomes that may be explained on the basis of high viscosity that decreased particle–particle collision and subsequent flocculation or fusion [18].

The aim of this study was to formulate TGS of ACZ as a double encapsulation strategy to investigate the effect of synergism between transfersomes as nano-carrier and in situ gel-forming solutions for prolonging drug release and improving the ocular delivery of ACZ by overcoming both the poor bioavailability of ACZ and the rapid drainage from the eye in the treatment of glaucoma.

## 2. Materials and Methods

### 2.1. Materials

ACZ was obtained as a gift from CID Pharmaceutical Company (Cairo, Egypt). Cremophor RH 40 and sorbitan monostearate (Span 60) were obtained from PureLab, USA. L-alpha-phosphatidylcholine (from soybean and egg yolk), Polyoxyethylene (20) sorbitan monolaurate (Tween 80), and polyoxyethylene (23) lauryl ether (Brij 35) were purchased from Sigma Chemical Co. (St. Louis, MO, USA). Sodium lauryl sulfate, potassium dihydrogen phosphate, and potassium monohydrogen phosphate were purchased from Alpha Chemika (Mumbai, India). Absolute ethanol was obtained from El-Nasr Pharmaceutical Chemical Company (Cairo, Egypt). Spectra/Pore^®^ dialysis membranes (12,000–14,000 Mwt cutoff) were obtained from Spectrum Laboratories, Inc. (Rancho Dominguez, CA, USA). All other chemicals and solvents were of analytical grade and were used as received.

### 2.2. Methods

#### 2.2.1. Preliminary Screening Studies

Preliminary screening studies were performed to evaluate the formulation parameters which may affect the properties of ACZ-loaded transfersomal nanovesicles (TNVs) and to identify the appropriate levels for different variables for optimization process. A total of 32 ACZ nanotransfersomal formulations were fabricated (Table 1 and Table 2). Two types of phospholipids (egg yolk PC and soybean PC) at different lipid-to-surfactant ratios and different types of edge activators, such as Brij 35, Tween 80, Cremophor RH, and Span 60, were employed for the preparation of the ACZ nanotransfersomal vesicles. Different ACZ-loaded TNVs were evaluated for their entrapment efficiency and physical appearance.

#### 2.2.2. Preparation of ACZ-Loaded TNVs

ACZ-loaded TNVs were fabricated by using the ethanol injection method due to its simplicity and reproducibility in producing nanovesicles [19,20]. The required quantities of PC, surfactants, and drug were dissolved in absolute ethanol (2 mL). The ethanolic solution was injected dropwise into a preheated aqueous phase (70 °C). Mixing of the formulated dispersion on a magnetic stirrer (Jenway 1000, Jenway, UK) was continued for another 1 h at room temperature, to ensure complete evaporation of any residual ethanol. The final volume of the nanotransfersomal dispersion was 10 mL. To prepare smaller vesicles, transfersomal dispersion was then sonicated at room temperature, using a bath sonicator (Elmasonic E 30 H, Elma, Singen, Germany) for three cycles of 5 min, with a rest of 3 min between cycles. The TNVs were left to mature overnight in a refrigerator at 4 °C to be used for further characterization.

#### 2.2.3. Experimental Design of ACZ-Loaded TNVs Using 3^2^ Factorial Design

According to the initial prescreening trials, the ratio of lipid to surfactant (X1) and type of surfactant (X2) were chosen as the critical formulation parameters for preparation of the target product with acceptable formulation characteristics and entrapment efficiency. ACZ-loaded TNVs were prepared according to a three-level (3^2^) factorial design, using Design-Expert software, Version 12 (Stat-Ease Inc., Minneapolis, MN, USA), to investigate the joint effect of formulation variables on the vesicle characteristics. In this design, the ratio of lipid to surfactant (X1) and type of surfactant (X2) were selected as independent variables, whereas the percentage of drug released after 8 h (Q_8h_, Y1) and entrapment efficiency (EE%, Y2) were chosen as dependent variables (Table 3) [21]. Each factor was screened at three levels (−1, 0 and +1) that labeled the lower level, the middle level, and the upper level, respectively.

Goodness of fit of this model to the experimental data was evaluated by using the coefficient of determination (R^2^), adjusted R^2^, and predicted R^2^. Furthermore, the results obtained from the factorial design were statistically analyzed, using analysis of variance (ANOVA). The significance level of each term was determined on the basis of F statistics and the *p*-value considering that the null hypothesis (H_0_) is true. A *p*-value less than 0.05 is considered significant at a level of significance of α = 0.05, indicating that the factor is significant and the null hypothesis can be rejected [22].

#### 2.2.4. Determination of EE% of ACZ-Loaded TNVs

The free (un-entrapped) drug was separated by ultracentrifugation at 15,000 rpm for 2 h at 4 °C, using a cooling ultracentrifuge (Biofuge, Primo Heraeus, Germany). The whole supernatant was analyzed for its drug content by using UV-Vis spectrophotometer at 266 nm (T80+, PG instruments Ltd., Leicestershire, UK). The EE% was calculated according to the following formula [23]:

(1) EE(%)=(A1−A2)×100/A1

where A1 = initial drug amount, and A2 = amount of drug determined in the supernatant.

#### 2.2.5. In Vitro Release Study of ACZ-Loaded TNVs

The solubility of ACZ was determined in distilled water and the dissolution medium, simulated lachrymal fluid (SLF) of PH = 7.4 and 1% sodium lauryl sulfate (SLS), using the shake-flask method [24] to ensure maintaining sink conditions of the dissolution medium.

The in vitro release study of ACZ-loaded TNVs was performed by using a modified Franz diffusion cell. The semi-permeable cellulose membrane was hydrated by using a SLF solution (PH = 7.4) for 24 h at 25 °C. The semi-permeable cellulose membrane was fixed between the donor and receptor chambers. The receptor medium was 20 mL SLF solution (PH = 7.4) and 1% SLS [25,26,27]. A SLF is used to simulate the in vivo conditions in order to attain a reasonable prediction for drug release [28]. SLS was added to maintain sink conditions [29]. The receptor compartment was kept under continuous stirring at 100 rpm, using a magnetic stirrer at 37 ± 0.5 °C. An accurate volume (1 mL) of transfersomal dispersions equivalent to 10 mg ACZ was placed in the donor chamber over the cellulose membrane. Then, 0.2 mL samples were taken at predetermined time intervals, and the receiver cell was replenished with an equal volume of fresh solution. The withdrawn samples were analyzed for drug content by spectrophotometric analysis at 266 nm. Three measurements were conducted for each study. The results were expressed as the mean values ± SD.

To determine the release kinetics and drug-release mechanism from ACZ-loaded TNVs, the drug-release data were treated by using different mathematical models, including zero-order kinetics, first-order kinetics, the Higuchi model, the Hixson–Crowell equation, and the Korsmeyer–Pappas model. The model with the highest coefficient of determination (R^2^) was chosen [30].

#### 2.2.6. Statistical Optimization of ACZ-Loaded TNVs

The desirability index was used for choosing the optimized formula. The desirability index investigates the desirable range for each response, and it is a value between 0 and 1. A desirability index of 1 expresses a completely desirable formula, while a desirability index of 0 expresses a completely undesirable formula [22]. In this model, the choice of the optimized formula was based on maximizing both Q_8h_ and EE%. 

The actual values of both responses (Y1 and Y2) were compared with the statistically predicted values and the % relative error was calculated, using the following equation, in order to validate the optimized formula [31]:

% Relative error = (predicted value − observed value) × 100/predicted value(2)

The optimized nanotransfersomal formula was evaluated by further characterization tests.

#### 2.2.7. Characterization of the Optimized ACZ-Loaded TNVs

##### Scanning Electron Microscopy (SEM)

The morphological characteristics of the optimized ACZ-loaded TNVs were investigated by using scanning electron microscopy (SEM) (JEOL, JSM-6360, Tokyo, Japan). The optimized transfersomal dispersion was suitably diluted with distilled water and mounted onto the aluminum stub, using double-sided sticking carbon tape. The sample was dried under vacuum and coated with a gold film. After coating, the TNVs were examined and photographed by SEM [32]. 

##### Zeta Potential Determination

The optimized transfersomal dispersion was diluted with distilled water. The zeta potential of the optimized nanotransfersomal dispersion was determined by detecting its electrophoretic mobility within an electrical field, using a NICOMP 380 ZLS zeta potential/particle sizer (PSS Nicomp, Santa Barbara, CA, USA), and the average of three replicates was recorded [33]. 

##### Measurement of Vesicle Elasticity

The elasticity and deformability of the optimized nanotransfersomal dispersion were evaluated by extrusion of the optimized nanotransfersomal dispersion (F7) from a nylon membrane filter with 100 nm pore size, at a constant pressure of 2.5 bar for 5 min [34]. The vesicle size of the optimized nanotransfersomal dispersion was determined before and after filtration. The deformability index was determined by using the following equation:

(3) $$D=J\;{\left(r_{v}/r_{p}\right)}^{2}$$

where *J* is the amount of the nanotransfersomal dispersion extruded, *r_v_* is the optimized nanotransfersomal vesicle size (after extrusion), and *r_p_* is the pore size of the nylon membrane filter.

The elasticity of the optimized nanotransfersomal dispersion (F7) was compared with the corresponding liposomal dispersion, to investigate the effect of the addition of EA.

##### Fourier Transform Infrared Spectroscopy (FT-IR) 

Potassium bromide pellets of the pure drug, different excipients, plain transfersomes, and the optimized transfersomal formulation were made in a hydraulic press (Kimaya Engineers, Maharastra, India), and then the pellets were scanned in the range of 4000–400 cm^−1^, using FTIR spectrometer (FT-IR Shimadzu 8300, Shimadzu Corporation, Kyoto, Japan) [35].

##### Differential Scanning Calorimetry (DSC)

DSC measurements of the pure drug, different excipients, plain transfersomes, and the optimized transfersomal formulation were carried out with a Shimadzu DSC 60 (Kyoto, Japan). Different samples were heated in aluminum pans, from 0 to 260 °C, with a scan rate of 10 °C/ min, and the DSC thermograms were recorded [36].

##### Effect of Storage on the Stability of the Optimized ACZ-Loaded TNVs 

Stability testing of different pharmaceutical products is performed to ensure the efficacy, safety, and quality of the active drug substance in the fabricated dosage forms during their storage. The optimized transfersomal formula was kept in a tightly closed clean container and stored in a refrigerator at 4 °C for 6 months. Samples were withdrawn at definite time intervals [37]. The optimized transfersomal formula was evaluated regarding its appearance, in vitro release profile, and EE%. The in vitro release profile of the optimized TNVs was compared to that of the freshly prepared formula, according to the similarity factor test in which in vitro release profiles are considered similar if the value of f2 lies between 50 and 100. Moreover, f2 is determined according to the following equation [30]:

(4) $$F2=50\times \log\left\{{\left[1+\frac{1}{n}\;\overset{n}{\underset{t=1}{\sum }}{\left(Rt-Tt\right)}^{2}\right]}^{-0.5}\times 100\right\}$$

where *Rt* and *Tt* are the percent of ACZ released at time, *t*, from the freshly prepared and the stored formulations, respectively, and n is the total number of sampling points.

#### 2.2.8. Formulation of the ACZ-Loaded TGS

ACZ-loaded TGS formulations were prepared by formulation of a thermoreversible gel of ACZ-loaded TNVs. ACZ-loaded TGS formula was formulated as a binary thermosensitive hydrogel, using 18% poloxamer 407 (Pluronic 127) and 5% poloxamer 188 (Pluronic 68) as gelling agents. The thermosensitive gel was prepared by the cold method [38]. Appropriate amounts of poloxamer 407 and poloxamer 188 were added slowly to cold water (4–5 °C), under constant stirring. The formulated dispersions were kept refrigerated until the formation of a clear solution. The transfersomal formula was added to the cold solution of polymer, with stirring, to obtain a homogeneous gel [39].

#### 2.2.9. Evaluation of the ACZ-Loaded TGS

##### Homogeneity

The homogeneity of the ACZ-loaded TGS was determined by visual inspection of the appearance of gel and the presence of any aggregates. This inspection was done by pressing a small quantity of gel between the thumb and the index finger [40].

##### Spreadability

The therapeutic efficacy of the gel is correlated with its spreadability. The spreadability of the fabricated gel was described by the extent of the area to which it spreads upon application to the affected part. A spreadability test of the ACZ-loaded TGS was performed by pressing 1 g of gel for 5 min between two glass slides, until no further spreading occurred. The average diameter of the formed circle was determined and used as a comparative parameter for the spreadability of gel [41].

##### pH

The pH of the ACZ-loaded TGS formulation was determined by using a digital pH meter (JENWAY, UK) after uniform dispersion of two grams of gel in distilled water (20 mL), using a magnetic stirrer [42].

##### Drug Content

The ACZ-loaded TGS formulation (1 g) was diluted to 100 mL with methanol and stirred for 2 h, using a magnetic stirrer, and was then allowed to stand for 24 h. The solution was then filtered and analyzed spectrophotometrically at 266 nm [43].

##### Gel Strength

The ACZ-loaded TGS formulation (25 g) was placed in a 100 mL graduated cylinder. Gelation was conducted by placing the cylinder in a water bath at 37 ± 1 °C, to simulate the physiological conditions [40,44,45,46]. A weight (10 g) is placed onto the gel and time required for penetration 5 cm into the gel is recorded and reported as the gel strength [47]. The gel strength measurement was also performed after diluting the formulation with SLF in a ratio of 40:7 in order to evaluate the change in gel strength after administration into the eye and mixing with the tear fluid [48]. The 40:7 ratio is chosen because the volume of the lacrimal fluid is 7 μL and the volume of the instilled ophthalmic drop is about 40 μL [49].

##### Measurement of Gelation Temperature (GT)

Ten milliliters of the sample solution was stirred on a magnetic stirrer, at minimal rpm. The temperature of the sample solution was increased gradually, at a rate of 1 °C/min. The temperature at which the movement of the magnetic bar stopped due to gelation was considered the gelation temperature [50].

The effect of dilution of ACZ-loaded TGS with SLF was investigated in order to mimic the in vivo phase transition of the in situ gels. The formulation was mixed with a SLF in a ratio of 40:7 before measurement [51].

##### Determination of Gelation Time and Gel Residence Time

First, 2 mL of the ACZ-loaded TGS formula was added to a test tube and kept in a water bath at 37 ± 0.5 °C, and the formation of gel was visually assessed, noting the time taken for the solution to convert into a gel (gelation time). The experimental temperature was kept at the 37 ± 0.5 °C, and the time taken for the formed gel to dissolve and lose its integrity (gel residence time) was recorded [52].

The effect of dilution of ACZ-loaded TGS with SLF on both the gelation time and the gel residence time was investigated by adding SLF in a ratio of 40:7 at 37 ± 0.5 °C [40].

##### Determination of the Rheological Properties

The viscosity of 3 g of the ACZ-loaded TGS formulation was measured before and after gelation at 25 °C ± 1°C and at 37 ± 0.5 °C, respectively, by a Brookfield R/S+RHEOMETER (rotary viscometer, Brookfield Engineering Laboratories, Inc., city, Middleboro, MA, USA), using spindle CC 14. The measurement was initiated at 1 rpm, and the speed was increased gradually until reaching 200 rpm. The speed was then decreased gradually, until reaching the starting rpm. The thixotropic behavior of the ACZ-loaded TGS formula was recorded, using a digital planimeter (KP-92n, Swastik Scientific Company, Mumbai, India), by calculation of the hysteresis loop between the upward and the downward curves of the transfersomal gel [53].

In addition, the effect of dilution of ACZ-loaded TGS with a SLF in a ratio of 40:7 was studied by measuring viscosity after dilution with a SLF at 37 ± 0.5 °C [40].

#### 2.2.10. In Vitro Release Study of ACZ-Loaded TGS

The in vitro release from the ACZ-loaded TGS formulation and ACZ aqueous dispersion through a semi-permeable cellulose membrane was determined by using a modified Franz diffusion cell, as described before. The in vitro profile of ACZ-loaded TGS was compared with the previous results of the corresponding transfersomal formula (F8), to investigate the effect of the addition of poloxamer polymers on the in vitro release profile of ACZ.

#### 2.2.11. Ex Vivo Corneal Permeation Study of ACZ-Loaded TGS

The ex vivo permeation study of ACZ-loaded TGS through the rabbit corneal membrane was performed and compared with that of ACZ-loaded TNVs (F8) and the aqueous dispersion of ACZ. Prior to performing the ex vivo corneal permeation study, the experiment protocol was approved by the ethical committee of the Faculty of Pharmacy, Kafrelsheikh University, Egypt (Approval number KFS-2018/09). Rabbit corneas used in this experiment were excised from white male New Zealand albino rabbits. The corneas were separated from the globes and washed with cold saline, followed by immersing in a SLF of pH 7.4 for 1 h before performing the experiment. The ex vivo corneal permeation study was conducted, using modified-Franz diffusion cells. The corneal membrane was fixed between the donor and receptor chambers in such a way that the corneal side remains in contact with the formulation. The receptor medium was SLF of PH = 7.4 and 1%SLS [25,26,27]. The receptor compartment was kept under continuous stirring at 100 rpm, using a magnetic stirrer at 37 ± 0.5 °C. An accurate volume of each formula (equivalent to 10 mg ACZ) was placed in the donor compartment over the corneal membrane. Samples were removed at the predetermined time intervals, and the receiver cell was replenished with an equal volume of fresh SLF at each time interval. The withdrawn samples were analyzed for drug content by spectrophotometric analysis at 266 nm. Three measurements were conducted for each study. The results were expressed as the mean values ± SD.

To determine the ex vivo permeation kinetics, the data were treated by using different mathematical models, including zero-order kinetics, first-order kinetics, the Higuchi model, the Hixson–Crowell equation, and the Korsmeyer–Pappas model. The model having the highest coefficient of determination (R^2^) was chosen [30].

To predict the mechanism of permeation of ACZ through the corneal membrane, the permeation rate of ACZ (flux, Jss) at steady-state was determined from the slope of the linear portion of the graphical plot of cumulative ACZ permeated through the corneal membrane versus time in steady-state conditions. The permeability coefficient (Kp) of ACZ was calculated by dividing the Jss on the initial concentration of ACZ. The enhancement ratio (ER) was determined by dividing Jss of the ACZ-loaded TGS or ACZ-loaded TNVs on Jss of ACZ aqueous dispersion [54].

#### 2.2.12. In Vivo Study of the ACZ-Loaded TGS

White male New Zealand albino rabbits, weighing 2.0–2.5 kg, were used in this experiment. The rabbits were obtained from the National Research Center (Dokki, Giza, Egypt) and housed in a pathogen-free facility in sawdust-bedded cages. The animal rooms were kept at 25 ± 2 °C, with 50% relative humidity, under a 12-h light and 12-h dark cycle. Acclimatization of the tested rabbits was performed in the animal house, under standard conditions, for at least two weeks before the experiment. Both the experimental protocol and animal used were approved by The Ethical Committee of Faculty of Pharmacy, at Kaferelsheikh University. All the performed procedures were in accordance with the ARRIVE guidelines, European Union Directive 2010/63/EU, and the UK Animals (Scientific Procedures) Act, 1986 (ASPA) [55].

##### Assessment of Ocular Irritancy

For assessment of the ocular tolerance of the fabricated ACZ-loaded TGS, rabbits were divided into 5 groups (3 rabbits each) for detection of any inflammation, increased tear production, or redness after ocular application. The first group was treated with fifty microliter aliquots of the ACZ-loaded TGS (equivalent to 500 µg of ACZ) into the conjunctival sac of the right eye. The second and the third groups were instilled with equivalent concentrations of ACZ-loaded TNVs and ACZ aqueous suspension, respectively. The fourth group was treated orally with an equivalent amount of commercial ACZ tablet (Cidamex^®^). The fifth group received no treatment and was kept as a control group. The eyes of the rabbits under investigation were examined visually, using a slit lamp [56].

##### Pharmacodynamic Study

The normal IOP of the tested New Zealand albino rabbits was 20.4 ± 1.9 mmHg. Ocular hypertension was induced by injecting 0.1% dexamethasone phosphate solution into the limbus of the right eye of different groups for 1 week [57,58]. The increase in IOP was monitored by using a Schiotz tonometer (Schiotz tonometer, Riester, Germany). The resting IOP in all animals was measured, using a tonometer, before drug administration. A 0.2% solution of Lidocaine HCI was used as a local anesthetic. Rabbits with high IOP were divided into five groups and treated with a single dose of each formulation (equivalent to 500 µg of ACZ), as previously described in the ocular irritancy test. The IOP was determined at 0.5, 1, 2, 3, 4, 6, 8, 12, and 24 h after drug administration [59]. Each measurement was repeated three times, and the data were expressed as the mean values ± SD.

##### Pharmacokinetic Studies in Aqueous Humor

New Zealand white rabbits (2.0–3.0 kg) were randomly divided into 3 groups (3 rabbits each) as animal model, to study the pharmacokinetics of ACZ in the aqueous humor. Each ACZ-loaded formulation, TGS, TNVs, and aqueous ACZ suspension (all contain 500 µg of ACZ) was treated with the lower conjunctival sac of the rabbit. The eyelids were kept gently closed for 10 s for maximizing the contact between the cornea and the drug [60]. After different time intervals, 50 μL sample of the aqueous humor was collected, using a 29-gauge insulin syringe. Aqueous humor samples were mixed with 100 μL HPLC mobile phase, a mixture of 0.01 M ammonium acetate and methanol (95:5), and then centrifuged at 4000 rpm for 15 min, and the supernatant was analyzed for drug content by HPLC technique at 266 nm [61], using a LPG-3400RS quaternary pump and an Inertsil reversed-phase C18 (150 × 4.6 mm × 5 μm). The eluent was monitored by using a DAD-3000RS diode array detector.

#### 2.2.13. Statistical Analysis

Statistical analysis was conducted by using SPSS-11 software (SPSS Inc., Chicago, IL, USA). All data were presented as the mean ± SD. The differences were considered statistically significant when *p* < 0.05.

## 3. Results

### 3.1. Preliminary Screening Studies

Transfersomes are ultra-flexible nanovesicular systems. These systems are mainly formulated by adding edge activators to the phospholipid bilayers of conventional liposomal vesicles. PC was used as the bilayer-forming agent.

Using biodegradable excipients with established safety profiles is of particular importance for fabrication of ocular dosage forms, to avoid irritation with the delicate tissues of the eye [62]. Non-ionic surfactants such as Tween 80, Cremophor RH 40, Span 60, and Brij 35 are FDA-approved excipients and included in FDA’s Inactive Ingredients Guide (FDA IIG) [63] and generally recommended as safe (GRAS) [64]. They find widespread applications in ocular drug delivery because they exhibited lower toxicity, lower ocular irritation, and better compatibility than anionic, cationic, and amphoteric surfactants [63,65]. In addition, they have a great tendency to maintain near the physiological pH [26]. Phospholipids such as PC are also approved by FDA and listed in FDA IIG [63] and GRAS [64]. They are commonly used in ocular drug-delivery systems such as liposomes, transferosomes, proniosomal gels, and pharmacosomes, because they have a wide safety margin, in addition to their biodegradability and biocompatibility [26,63].

In the present study, PC is considered to be the main component of transfersomes and used in the range of 60–90 mg. Span 60, Tween 80, Cremophor RH 40, and Brij 35 were used as non-ionic surfactants in the range of 10–40 mg. Previous studies, such as one by Janga et al., prepared transfersomes, to improve the ocular delivery of natamycin, using 23–52 mg of Span 60 and 91–136 mg of Phospholipid 90H [66].

Other researchers used these excipients for the fabrication of different ocular drug-delivery systems but at higher concentrations. However, they reported the absence of any sign of ocular inflammation. Abdelbary et al. [26] have used Span 60, Tween 80, and Brij 35 at concentrations of 250 and 500 mg, in addition to 250 mg of PC for preparation of ocular ketoconazole-loaded proniosomal gels and observed absence of any sign of inflammation over the study period. In addition, Eldeeb et al. [67] utilized Span 60, Brij 52, and Tween 80 in the range of 180–540 mg for the fabrication of proniosomal ocular gel of Brimonidine tartrate and used lecithin in the range of 180–360 mg. Moreover, Tiwari et al. [68] formulated an ocular self-microemulsifying drug-delivery system of Prednisolone, using 55–65% of Cremophor RH 40, which exhibited the absence of any ocular inflammation.

The preliminary screening study was conducted to select the levels of the factors considered for the experimental design on the basis of entrapment efficiency. With respect to the type of phospholipid, ACZ-loaded TNVs produced using soybeans PC were found to be more uniform than egg yolk PC. These results are in agreement with Guldiken et al. [69]. In addition, EE% of ACZ-loaded TNVs prepared by soybean PC ranged from 31.51 ± 1.58% to 97.30 ± 1.68, while EE% of ACZ-loaded TNVs prepared by egg yolk PC ranged from 28.61 ± 1.33% to 94.60 ± 1.74%. Although EE% was higher in case of soybean PC, no significant variation (*p* > 0.05) in EE% was noticed between nanotransfersomal formulations prepared by both types of phospholipids. Therefore, the type of phospholipid was not chosen as an independent variable and soybean PC was selected for the fabrication of ACZ-loaded TNVs.

Concerning the lipid to surfactant ratio, it was found that the EE% of ACZ-loaded TNVs at 60:40 lipid-to-surfactant ratio was significantly (*p* < 0.05) lower than the other ratios, which may be attributed to the formation of mixed micelles with a low drug encapsulation capacity [70] and destabilization of the phospholipid bilayer at high surfactant concentrations [71].

Regarding the type of surfactant, ACZ-loaded TNVs containing Brij 35 showed the lowest EE% compared to those containing Span 60, Cremophor RH, and Tween 80. That may be attributed to the higher HLB value of Brij 35 (16.9) and the shorter alkyl chain length (C12) chain compared to the other surfactants [72].

Thus, considering EE%, two independent variables were chosen for the optimization process: the ratio of lipid to surfactant (X1) and the type of surfactant (X2). Soybean PC was chosen as the bilayer-forming agent at lipid-to-surfactant ratios 90:10, 80:20, and 70:30. Tween 80, Span 60, and Cremophor RH were selected as the edge activators.

### 3.2. Analysis of Factorial Design

The purpose of the optimization process is to determine the levels of variables that are required for the fabrication of high-quality products. Design of experiments (DOE) is a technique used to plan experiments and analyze the information obtained, in order to evaluate which variable(s) are the most important contributors to any process from a set of variables. The technique allows us to use a minimum number of experiments in which we systematically vary a number of independent variables to study their effect on different responses [73].

For the formulation of ACZ-loaded TNVs, the two independent variables, the ratio of lipid to surfactant (X1) and type of surfactant (X2), were screened by a (3^2^) factorial design in which the two factors (X1 and X2) were evaluated at three levels (Table 4). The optimized formulation of the ACZ-loaded TNVs was selected on the basis of achieving the maximum Q_8h_ (Y1) and maximum entrapment efficiency (Y2).

In the present model, the values of R^2^ for both the percentage of drug released after 8 h (Q_8h_, Y1) and entrapment efficiency (EE%, Y2) are relatively high, 0.9669 for Y1 and 0.8973 for Y2, indicating that the obtained data are highly statistically valid. The predicted R^2^ values were determined to investigate the response value predictability of the model. The adjusted R^2^ compensates for the addition of variables to the model. As more independent variables are added to the regression model, the R^2^ value will increase. However, the adjusted R^2^ decreases or increases depending on whether the additional variables detract or add to the explanatory power of the model. Therefore, the adjusted R^2^ is generally considered more accurate than R^2^, and its value might be lower or equal to that of R^2^ [74]. In this model, for responses (Y1 and Y2), the adjusted R^2^ value was less than the R^2^ value.

Table 5 shows that the predicted and adjusted R^2^ values of different responses were in acceptable agreement because the difference between them was less than 0.2. The signal-to-noise ratio is determined by adequate precision. Adequate precision was found to be more than the desired value (4) for Y1 and Y2. Consequently, this model is appropriate for navigating the design space [75].

ANOVA (Table 6) illustrated the significance of different factors. The model terms are considered significant when the *p*-value is lower than 0.05. Accordingly, H_0_ (the null hypothesis) is rejected, and the alternative hypothesis is accepted.

### 3.3. The Effect of Formulation Variables on Q_8h_ of ACZ-Loaded TNVs

The sink condition is a critical requirement for the in vitro release studies. Sink conditions could be described as at least three times the volume required for obtaining a saturated solution of the drug. The addition of a surfactant, such as SLS, to the dissolution medium is important to ensure sink conditions for the poorly soluble drugs [76]. The solubility of ACZ in distilled water was found to be 0.82 ± 0.61 mg/mL, whereas its solubility in the SLF of PH = 7.4 and 1% sodium lauryl sulfate was 1.93 ± 0.43 mg/mL.

**Figure 1:**
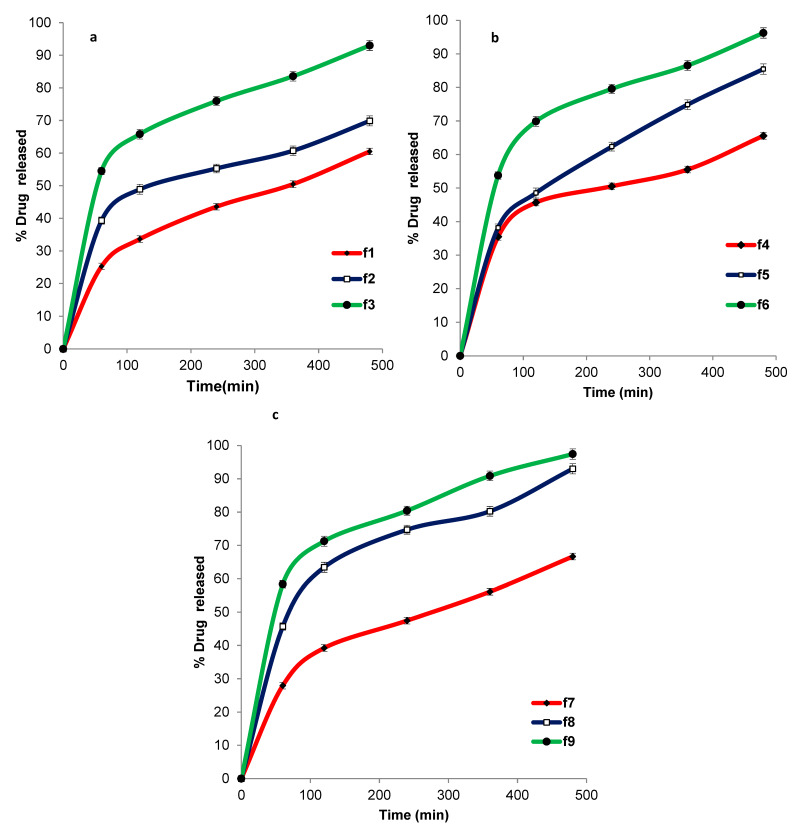
In-vitro release of ACZ-loaded TNVs containing Span 60 (**a**), Tween 80 (**b**), and Cremophor RH (**c**) as edge activators.

Figure 1 shows the release profile of ACZ from different transfersomal formulations that ranged from 56.75 ± 0.82 to 97.43 ± 1.89. The improved release of ACZ from the transfersomal vesicles may be attributed to the softness and deformability of the transfersomal membrane due to the presence of surfactant that acts as an edge activator or bilayer softening agent [77]. Moreover, the highly hydrophilic transfersomal vesicles always seek to avoid dehydration by migration to the water-rich layers, which is considered a transport process that is related to the osmotic gradient [78].

**Figure 2:**
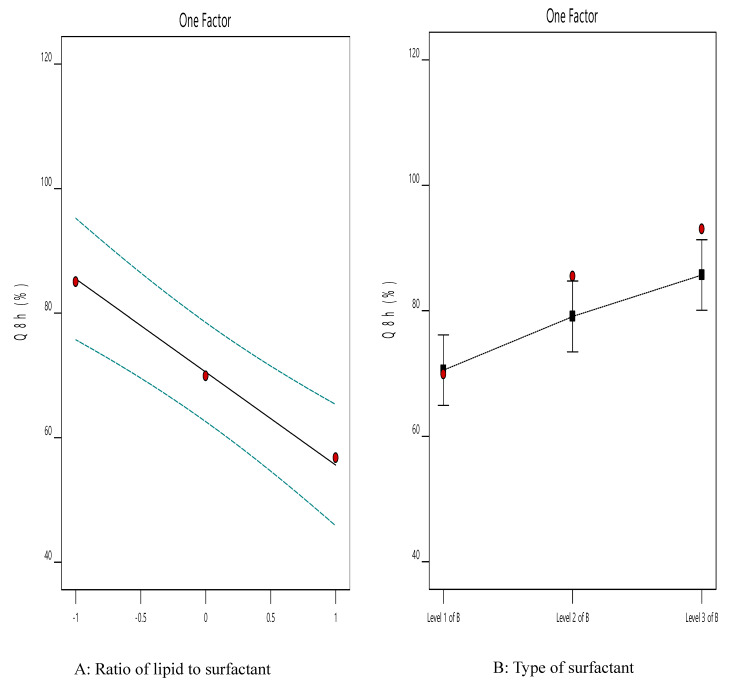
The effect of different independent variables on Q_8h_ of ACZ-loaded TNVs.

Figure 2 illustrates the effect of the independent variables on Q_8h_ (Y2). It is clear that the highest Y1 value was observed in the ACZ nanotransfersomes that included cremophor RH as the edge activator and a 70:30 lipid-to-surfactant ratio (F9).

The ANOVA results (Table 6) indicated that both factors had a significant impact on the Q_8h_ of the prepared TNVs. The lipid-to-surfactant ratio (X1) had a significant influence (*p* < 0.001) on Q_8h_. It is obvious that Q_8h_ increased at higher edge activator ratios, which may be explained by the fact that increasing the concentration of surfactant leads to better solubilization of drug and, therefore, higher drug release [79]. Moreover, this effect may be due to decreasing the encapsulation efficiency of nanovesicles at higher concentrations of surfactant and lower concentrations of lipid, leading to better efflux of the entrapped drug [80].

These results are in accordance with Pitta et al., who reported that the drug release from the transfersomal vesicles is affected by the composition and characteristics of their ingredients in addition to the difference in their entrapment efficiency [12].

The drug-release profile of ACZ was also significantly (*p* < 0.05) related to the type of surfactant (X2). Transfersomes containing Cremophor RH achieved higher drug release than those containing Span 60 and Tween 80, which may be attributed to the better solubilizing capacity of Cremophor RH [79,81,82].

A drug-release kinetic study (Table 7) demonstrated that the correlation coefficient values (R^2^) were highest for the Higuchi model, indicating that the drug release from all the nanotransfersomal formulations was best fitted by the diffusion-controlled release mechanism.

### 3.4. The Effect of Formulation Variables on EE% of ACZ-Loaded TNVs

Entrapment efficiency is a vital tool for determining the stability of nanovesicles [83]. The ACZ-loaded TNVs showed good EE% that ranged from 62.39 ± 1.55 to 97.30 ± 1.68 (Table 4). Clearly, the highest Y2 value was observed from the ACZ-loaded nanotransfersomes containing Span 60 and a 90:10 lipid-to-surfactant ratio (F7).

**Figure 3:**
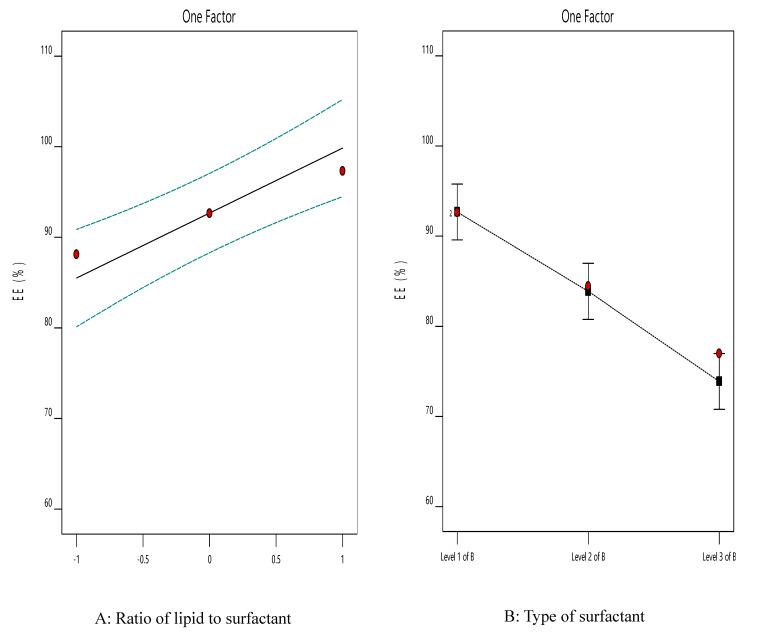
The effect of different independent variables on EE% of ACZ-loaded TNVs.

Figure 3 shows the influence of different independent variables on the EE% of ACZ-loaded TNVs. The statistical analysis (Table 6) revealed that both the lipid-to-surfactant ratio (X1) and the type of surfactant (X2) had a significant effect on EE% (*p* < 0.05). It is obvious that the encapsulation efficiency increased at higher phospholipid concentrations, which may be attributed to the presence of a higher number of transfersomal vesicles and hence larger transfersomal core [84]. In addition, as the surfactant concentration increased, the encapsulation efficiency of the TNVs decreased, which might be due to mixed micelle formation with a low drug loading capacity [70]. Moreover, high concentrations of surfactant lead to destabilization of the phospholipid bilayer and pore formation, thus decreasing the entrapment efficiency [71].

Concerning the type of surfactant (X2), transfersomes containing Span 60 have higher encapsulation efficiency than those containing Cremophor RH and Tween 80. These results may be attributable to the higher affinity of the surfactant to phospholipid in the case of Span 60 (HLB = 4.3) than in the case of Tween 80 (HLB = 15) and Cremophor RH (HLB = 14–16) due to the higher lipophilicity of Span 60, which in turn caused higher vesicle rigidity [85]. These results are consistent with other studies, such as those by Khalil et al. [86] and Lv et al. [87].

The EE% was higher in transfersomal formulations containing Cremophor RH than in those containing Tween 80, which may be explained by the fact that the structure of Cremophor features a saturated non-branched chain that could result in stabilization of the nanovesicles and better drug entrapment. In comparison, Tween 80 showed a more steric structure. Therefore, at higher concentrations of Tween 80, there was no more area on the vesicle surface to be occupied [71]. These results were also shared by other researchers, such as Petchsomrit et al. [79].

**Figure 4:**
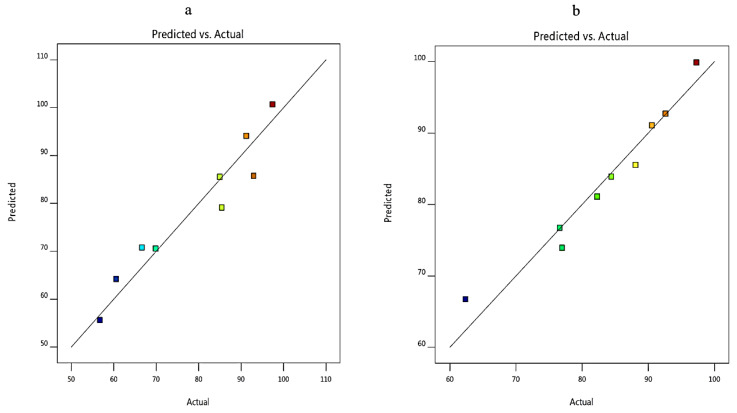
Linearity plots of ACZ-loaded TNVs shown as observed versus predicted values (**a**) Y1 and (**b**) Y2.

The linear correlation plots between the observed and the predicted values of Q_8h_ (Y1) and EE% (Y2) of ACZ nano-transfersomes were illustrated in Figure 4. The observed values were determined from the experimental results, and the predicted values were derived from the mathematical equations. It was observed that there was an excellent fit between the observed and predicted values of Y1 and Y2 in this model.

The predicted responses for the optimized formula (F8) were found to be 90.63% and 81.77% for Y1 and Y2, respectively. The % relative error was found to be less than 5 (−2.62 and −3.27 for Y1 and Y2, respectively), indicating the fitness of the model [31].

### 3.5. Optimization of the ACZ-Loaded TNVs

**Figure 5:**
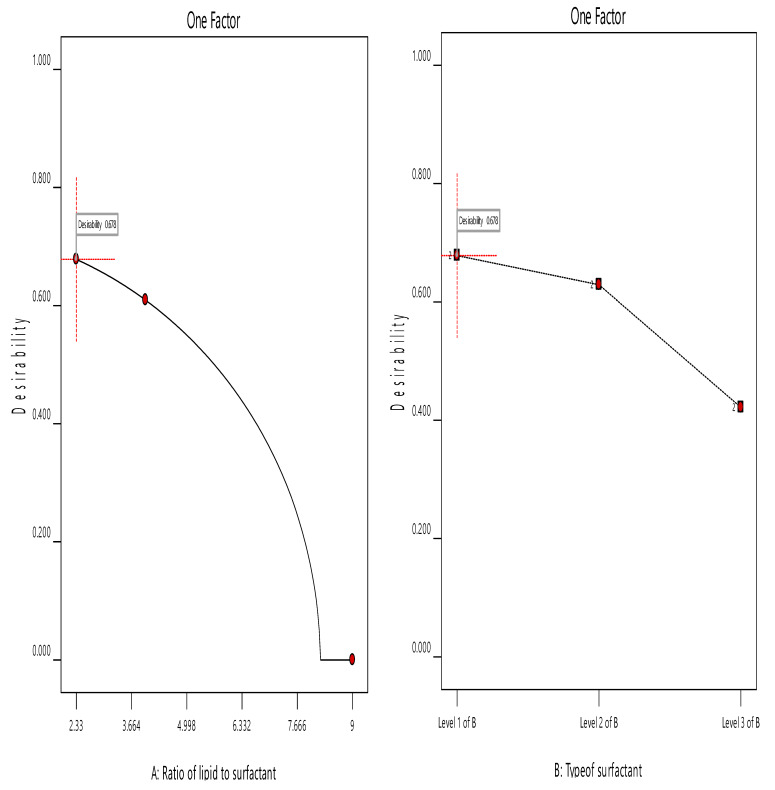
The overall desirability of ACZ-loaded TNVs as a function of independent variables (**a**) the ratio of lipid to surfactant and (**b**) the type of surfactant.

The choice of the optimized formula was based on the desirability criteria (Figure 5) through numerical and graphical optimization techniques. In the optimization study, the estimation of different desirability functions is based on the chosen criteria (maximize, minimize, target value, or have a certain range) for each response. The desirability approach could transform the estimated response (Y1 and Y2) into a desirability value, which increases as the value of the corresponding response becomes more desirable. The desirability value ranges between 0 and 1. A desirability value of 1 expresses a completely desirable formula, while a desirability value of 0 expresses a completely undesirable formula. The formula that has the highest desirability value is selected as the optimized formula [34,88].

The ACZ-loaded TNVs were optimized on the criteria of attaining maximum drug release and maximum entrapment efficiency. F8, composed of PC and Cremophor RH at ratio of 80:20, had the highest desirability index (0.678); thus, it was chosen as the optimized formulation.

### 3.6. Characterization of the Optimized ACZ-Loaded TNVs

#### 3.6.1. Vesicle Size and Zeta Potential Determination

**Figure 6:**
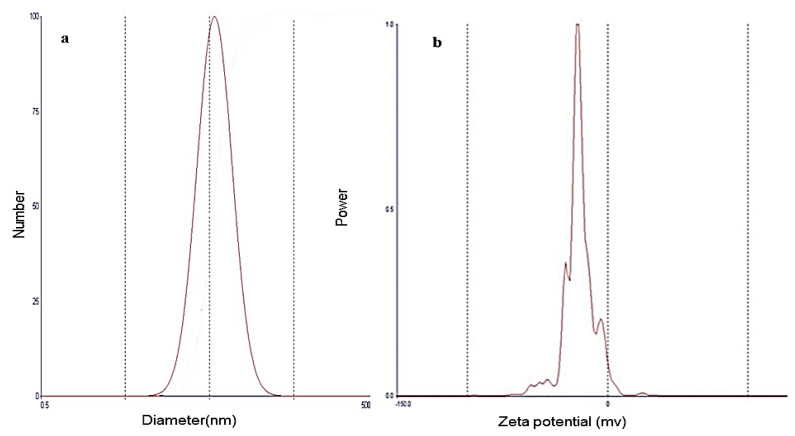
Particle size distribution curve (**a**) and zeta potential (**b**) of the optimized ACZ transfersomal formula (F8).

The optimized ACZ nanotransfersomal formula was evaluated for particle size, zeta potential, and polydispersity index (PDI). The results showed that the vesicular size of the optimized formulation was 203.4 ± 0.8 nm. The uniform size distribution of the nanotransfersomal vesicles is expressed by PDI. The closer to zero the PDI value is, the higher the homology between the particles. The PDI value of the optimized ACZ nanotransfersomal formula was 0.279 (less than 0.5), indicating a narrow and homogenous size distribution. The particle size distribution showed symmetric and unimodal frequency distribution pattern (Figure 6). Zeta potential is defined as the potential at the hydrodynamic shear plane of colloidal dispersions. Large values of zeta potential (positive or negative charges) reflect the stability of colloidal dispersions [89]. The optimized ACZ-loaded transfersomal formula has a zeta potential value of −19.20 mv, indicating the stability of the optimized transfersomal formulation. This stability may be attributable to repulsion between the nanovesicles that prevents their agglomeration, providing a highly stable and uniformly dispersed suspension [90]. The above results indicated the formation of uniform nanovesicles with high physical stability.

#### 3.6.2. Morphological Characterization by SEM

**Figure 7:**
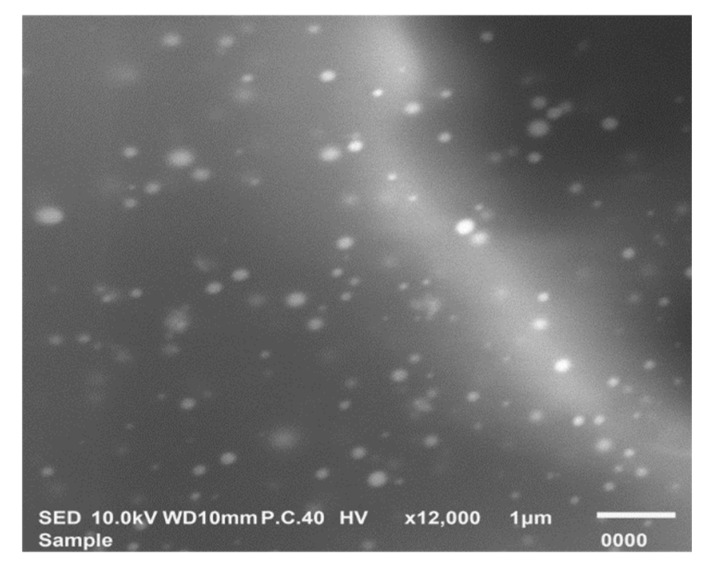
Scanning electron micrograph of the optimized ACZ transfersomal formula (F8).

The morphology of the optimized ACZ-loaded transfersomal formula was examined by SEM (Figure 7). Nano-transfersomal vesicles appeared as a homogenous dispersion of well-identified spherical vesicles.

#### 3.6.3. Measurement of Vesicle Elasticity

The deformability and elasticity of the transfersomal vesicles are critical parameters that distinguish them from the conventional liposomal vesicles. The deformability of the optimized ACZ-loaded transfersomal dispersion was investigated by measuring its ability to pass through pores that are smaller in size than their own diameter. The deformability index value (DI) of the optimized ACZ-loaded transfersomal dispersion (18.22 ± 1.4) was significantly (*p* < 0.001) higher than that (0.83 ± 0.04) of the corresponding liposomal dispersion.

High deformability and elasticity of ACZ nanotransfersomal vesicles reflect their ability to penetrate through the biological membranes, without losing their vesicle integrity, which could be attributable to incorporating non-ionic surfactants that destabilize the lipid bilayer, increasing its deformability and elasticity [91].

#### 3.6.4. Fourier Transform Infrared (FTIR) Spectroscopy

**Figure 8:**
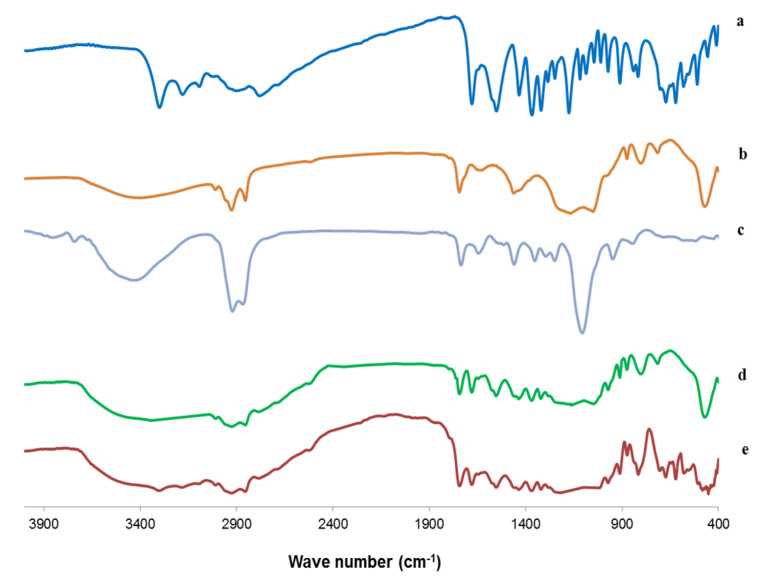
FTIR images of (**a**) ACZ, (**b**) PC, (**c**) Cremophor RH, (**d**) the plain transfersomes, and (**e**) optimized transfersomal formula.

Drug-excipient interactions were studied by using FTIR spectroscopy. The FTIR spectra of ACZ, PC, Cremophor RH, the plain transfersomes, and the optimized transfersomal formula (F8) are depicted in Figure 8. The FTIR spectrum of PC exhibited C=O stretching vibration at 1730 cm^−1^; a bending vibration of –CH3 deformation at 1418 cm^–1^; C=C asymmetric stretching of bending vibration at 700 cm^–1^, bending vibration of –CH3 deformation at 1430 cm^–1^, PO_2_ vibration at 1166.24 cm^−1^; and P-O-C vibration at 1035.10 cm^–1^ [92,93].

The FTIR spectrum of Cremophor RH showed a broad absorption band at 3386 cm^−1^ due to the OH group; bi-forked peak with two apices at 2856 and 2904 cm^−1^ due to the aliphatic –CH bond; a band at 1725 cm^−1^ corresponding to the carbonyl group of ester; and a strong broad band at 1093 cm^−1^ due to the C–O–C stretching vibration [94].

The FT-IR spectrum of ACZ exhibited characteristic peaks at 3296 and 3174 cm^−1^ attributable to N–H stretching of the secondary amine. The band present at 1678 cm^−1^ corresponded to the C=O stretching of the carboxyl groups. S=O stretching of sulfonyl group was observed at 1173 cm^−1^. The characteristic absorption band at 907 cm^−1^ was attributed to the S–N stretching [95]. The FTIR spectrum of the plain (drug-free) transfersomal formula showed the characteristic peaks of both PC and Cremophor RH. Moreover, the IR spectrum of the optimized nanotransfersomal formula (F8) displayed the characteristic peaks of ACZ and different excipients, which showed the absence of any chemical interactions between ACZ and the excipients [30].

#### 3.6.5. Differential Scanning Calorimetry (DSC) Study

**Figure 9:**
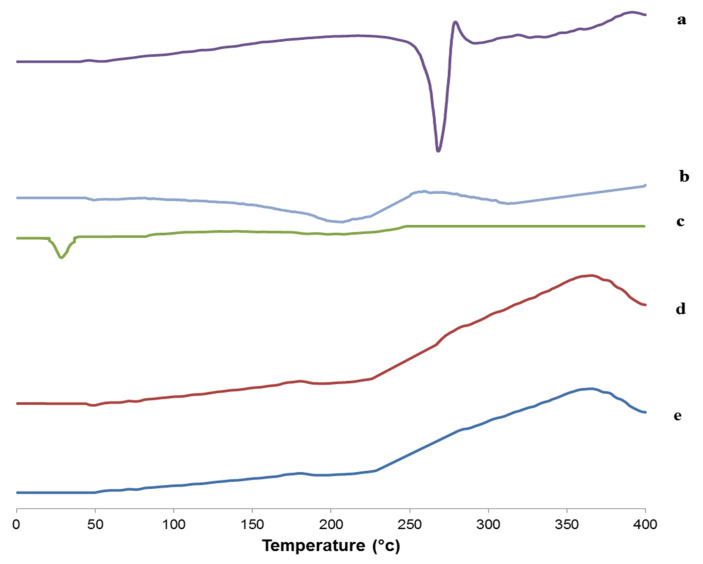
DSC thermogram of (**a**) ACZ, (**b**) PC, (**c**) Cremophor RH, (**d**) the plain transfersomes, and (**e**) optimized transfersomal formula.

The thermal behavior and the physical state of the drug could be detected through thermal analysis. DSC thermograms of ACZ, PC, Cremophor RH, the physical mixture, and the optimized transfersomal formula (F8) are presented in Figure 9. The DSC thermogram of ACZ showed an endothermic peak at 267 °C that revealed the crystallinity of ACZ [94]. The DSC of PC exhibited a characteristic endothermic peak at 203.7 °C that may be due to the isotropic liquid phase of the phospholipid [96]. The DSC thermogram of Cremophor RH showed a peak at 26.1 °C corresponding to its melting transition [94,97]. The DSC thermogram of the plain transfersomes exhibited a broad peak at 222.4 °C, which indicated the interaction of the components of the nanotransfersomal vesicles during the development of the lipid bilayer [98]. Interestingly, an analogous peak was observed from the chosen nanotransfersomal formula at 224.9 °C, and the endothermic peak of ACZ completely disappeared, and this could be attributed to the perfect encapsulation of ACZ in the transfersomal vesicles and the dispersion of ACZ as an amorphous state in the nanovesicles increasing the phase transition temperature [5].

#### 3.6.6. Effect of Storage on the Stability of the Optimized ACZ-Loaded TNVs

The effect of storage on the stability of the optimized ACZ-loaded TNVs was studied for six months at 4–8 °C (Table 8). No change in the physical appearance of the transfersomal formula was detected. In addition, there was no significant difference in the Q_8h_ and EE% of the stored transfersomal formula when compared with the fresh formula (*p* > 0.05). Moreover, the similarity factor test showed an insignificant difference in the in vitro release profile of the optimized transfersomal formula after storage (f_2_ was found to be 75).

### 3.7. Formulation of ACZ-Loaded TGS

Poloxamers are FDA-approved excipient and included in FDA IIG [63] and GRAS [64]. They are water-soluble convenient choices in the pharmaceutical formulations due to their wide range of molecular weights, availability, peculiar behavior, and high flexibility [99]. Besides, poloxamers have an amphiphilic character, demonstrate surface-active properties, and are able to interact with biological membranes and hydrophobic surfaces. They are also biodegradable, non-toxic, and non-irritant to the corneal mucosa; they also do not interfere with normal vision due to their transparency and exhibit reverse thermal gelling property [100]. Hence, poloxamers are appropriate for use in ocular drug delivery. Both Poloxamer P407 and Poloxamer 188 are the most commonly used that may be attributable to their good water solubility, optimum viscosity, and ocular tissue safety [101].

The reverse thermal gelling property of poloxamers means that poloxamer solution forms a clear liquid at a cold temperature (4–5 °C) and forms a gel at body temperature [102]. The reverse thermal gelling property may be due to a change in the micellar number with increasing temperature. In addition, hydrogen bonds between water and poloxamer chains keep the hydrophobic portions of the poloxamer separate in cold water. However, increasing the temperature causes disruption of the hydrogen bonds, and the hydrophobic interactions cause the micelles to be tightly packed such that the solution becomes immobile, leading to gel formation [17].

The optimized ACZ-loaded transfersomal formula (F8) was formulated as a thermosensitive TGS, using 18% poloxamer 407 and 5% poloxamer 188 as gelling agents. Poloxamer 407 is transformed from a low-viscosity solution to a gel at a concentration of 18% (*w*/*w*) or higher at ambient temperature. Therefore, poloxamer 188 was added as a regulatory substance in order to increase the GT of the binary formulation to the physiological range, and hence we could use the lowest possible concentration of poloxamer 407. Moreover, poloxamer 188 prevents the loss of the gelation ability of poloxamer 407 after dilution by the lacrimal fluid in the eye [40].

Other researchers used higher concentrations of poloxamer P407 for ocular drug delivery and reported the absence of any ocular inflammation, such as Al Khateb et al. [100], who reported that in situ gelling systems containing 20% *w*/*w* poloxamer P407 exhibited no ocular irritation. In addition, Fathalla et al. [103] studied the blend of poloxamers P407 and poloxamer 188 for preparing thermoresponsive ketorolac tromethamine (KT) in situ gel, to achieve controlled ocular delivery of KT. The most promising gel formulations were those containing 23:10 *w*/*v*% and 23:15 *w*/*v*% of Poloxamer 407:Poloxamer 188, respectively. These gels were found to be non-irritant to the conjunctiva and cornea.

### 3.8. Evaluation of ACZ-Loaded TGS

The ACZ-loaded TGS was subjected to characterization by evaluating various parameters, such as homogeneity, spreadability, pH, drug content, gel strength, gelation temperature, and rheological properties. It was found that the ACZ-loaded TGS has a smooth and homogenous appearance and was free of any particulate matter. ACZ-loaded transfersomal has a good spreadability value of 12.3 ± 0.54 cm, which showed that it can be spread easily on the skin surface with little stress. The pH of the ACZ-loaded TGS was found to be 7.1, which was within the physiological range of the eye (7 to 7.4); hence, it would not cause any irritation upon administration [104]. The drug content was found to be 98.82 ± 2.15%, which shows good content uniformity.

The gel strength is important for the maintenance of gel integrity. The gel strength observed for ACZ-loaded TGS was 65 ± 0.48 s. The high gel strength value may be due to the addition of poloxamer 188 to poloxamer 407 [40]. The addition of SLF had no significant effect (*p* > 0.05) on the gel strength. The gel strength after dilution with SLF was 63 ± 0.82 s that exhibited the stability of TGS upon dilution with SLF into the eye.

The GT of the ACZ-loaded TGS was found to be 37.2 ± 0.6 °C. Therefore, ACZ-loaded TGS could convert to a gel at physiological temperature. Gelation of in situ gel at lower temperatures will cause difficulty in gel administration, while gelation at higher temperatures will cause rapid drainage by tear fluid [105]. The GT, after dilution with the SLF, is 37.5 ± 0.3 °C. Hence, no significant change (*p* > 0.05) was detected in GT upon dilution with SLF, showing the ability of TGS formulation to remain as the gel form upon dilution with tear fluid.

The gelation time is the time required for an in situ gel for the transition from sol to gel state. An ideal formula should convert to gel immediately on exposure to the physiological temperature. The gel residence time corresponds to the time until which the formed gel remains intact. This shows the integrity of the formed implant matrix to provide a sustained release the desired period of time [52]. ACZ-loaded TGS exhibited immediate gelation with gelation time of 4 ± 0.8 s and remained intact for a prolonged period of time for 24 ± 0.8 h, showing an optimum gelling capacity, so that after application into the eye, it would undergo a rapid sol-to-gel transformation and would preserve its integrity for an extended period of time. After dilution with SLF, the gelation time and gel residence time were 4.7 ± 0.9 s and 23.3 ± 0.5 h, respectively. Hence, no significant effect (*p* > 0.05) of SLF on both the gelation time and gel residence time investigating the stability of TGS after dilution with the lachrymal fluid in the eyes.

An ophthalmic formulation should be characterized by both easy instillation into the eye as liquid droplets and rapid sol–gel transformation in the eye to achieve a prolonged effect. The ideal viscosity of ophthalmic solutions, that permits the easy application of the formulation ranges from 25–50 CP [106,107]. The viscosity of ACZ-loaded TGS before gelation at 25 °C ± 1 °C lies within this acceptable range and thus permits easy administration into the eye. The minimum and maximum viscosities of ACZ-loaded TGS, before gelation, are 21.38 and 40.12 CP, respectively.

**Figure 10:**
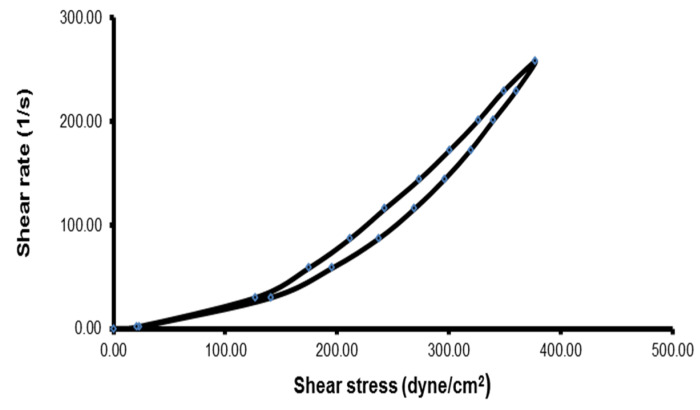
Thixotropic behavior of ACZ-loaded TGS (up and down curves) at 37 ± 0.5 °C.

In addition, there was an increase in the viscosity of ACZ-loaded TGS after gelation at 37 ± 0.5 °C that increases the contact time on the corneal surface. That could be explained on the basis of increasing the micellar number at a higher temperature [40]. The rheological behavior of ACZ-loaded TGS formulation is depicted in Figure 10.

The in situ gel exhibited pseudo-plastic flow (decreasing viscosity with increasing shear rate), which is evidenced by the fact that the flow curves approach the origin with no yield values and that the N value is higher than 1 (Table 9). This shear-thinning behavior is responsible for the uniform distribution of drug on the corneal surface of eye. Moreover, the transfersomal gel showed good thixotropic behavior, which is recommended for attaining prolonged residence in the lachrymal fluid by increasing the contact time of the in situ gel on the corneal surface [40].

However, no significant effect (*p* > 0.05) of the addition of SLF on the rheological behavior of ACZ-loaded TGS confirming the ability of TGS to remain stable into the eye after dilution with the lachrymal fluid (Table 9).

### 3.9. In Vitro Release Study of ACZ-Loaded TGS

**Figure 11:**
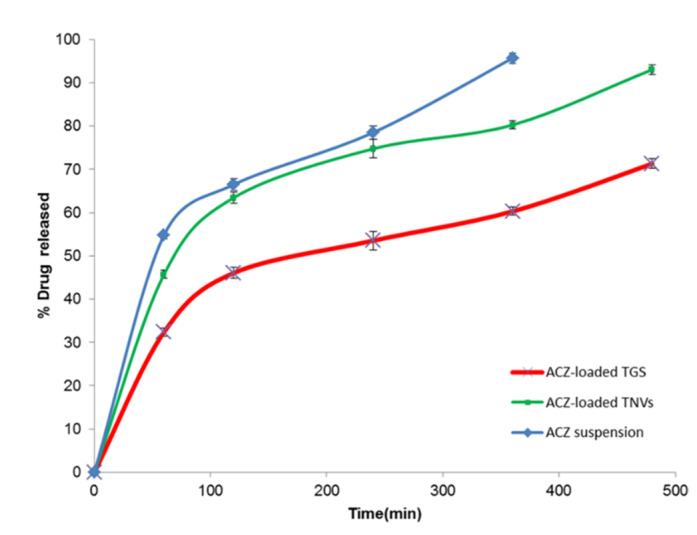
In vitro release profile of ACZ-loaded TGS, ACZ-loaded TNVs, and ACZ aqueous dispersion (*n* = 3).

The in vitro release of ACZ-loaded TGS in comparison with that of ACZ-loaded TNVs (F8) and ACZ aqueous dispersion is depicted in Figure 11. The Q_8h_ of ACZ-loaded TGS was found to be 71.28 ± 0.46%. However, 95.68 ± 1.41% of the free drug was released after 6 h.

It is obvious that the release of the aqueous dispersion is higher than that from ACZ-loaded TGS, which may be attributable to the fact that the release pattern of ACZ from the TGS is a combination of drug release from transfersomal vesicular system and diffusion through the cross-linked network structure of the poloxamer gel [108]. In addition, the higher % drug released of free drug of control confirms that the sink conditions were accomplished and the cellulose membrane used did not hinder the in vitro release of ACZ. These results are in accordance with other researchers, such as Moawad et al. [109], who reported that the release of free drug was markedly faster than that from transfersomal vesicles.

In addition, ACZ-loaded TNVs (F8) demonstrated a higher Q_8h_ of 93.01 ± 3.76% than that of ACZ-loaded TGS. These results reflect the role of poloxamer-based gel in prolonging the drug release that permits longer contact time with the corneal area.

Table 10 shows the kinetic study of the in vitro release of the ACZ-loaded TGS and ACZ aqueous dispersion according to the correlation coefficient values [110]. The results demonstrated that the release from the ACZ-loaded TGS and ACZ aqueous dispersion followed Higuchi model kinetics and zero order kinetics, respectively.

### 3.10. Ex Vivo Corneal Permeation Study of ACZ-Loaded TGS

**Figure 12:**
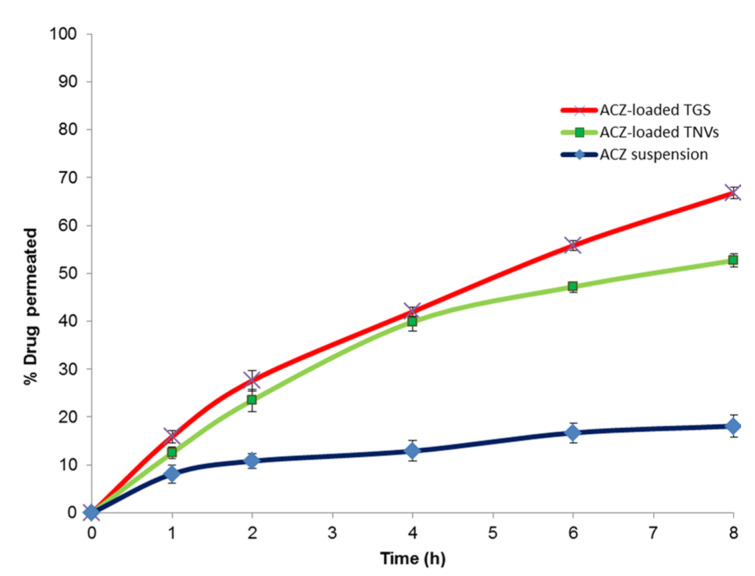
Ex vivo corneal permeation profile of ACZ-loaded TGS, ACZ-loaded TNVs, and ACZ aqueous dispersion (*n* = 3).

Figure 12 illustrates % ACZ permeated through the isolated corneal membrane from ACZ-loaded TGS, ACZ-loaded TNVs (F8), and ACZ aqueous dispersion. It is clear that there was a significant increase in the permeation of ACZ from ACZ-loaded TGS and ACZ-loaded TNVs compared to the free drug (*p* < 0.05).

The ex vivo corneal permeation of ACZ aqueous dispersion was 18.10 ± 1.34% after 8 h, whereas the % ACZ permeated from TNVs was 52.70 ± 1.42%. ACZ-loaded TGS exhibited higher ex vivo permeation value than TNVs reaching 66.82 ± 1.11% after 8 h. Furthermore, ACZ-loaded TGS showed higher steady-state flux and permeability coefficient values (Table 11) relative to both ACZ-loaded TNVs and ACZ dispersion, with an enhancement ratio of 7.86.

The enhanced permeation of ACZ-loaded TGS than ACZ aqueous dispersion could be attributable to the flexibility and elasticity of the nanotransfersomal vesicles that assist ACZ permeation through the corneal membrane relative to the free ACZ, due to presence of edge activators [111]. In addition, phospholipids have a high affinity for the biological membranes that lead to improvement of the permeation efficiency [109]. Moreover, the pluronic-based gel has unique characteristics, including the thermoreversible gelation and micellar properties. The pluronic-based gel could improve drug permeation via diffusion through the lipid intracellular matrix by a slight disorganization of the biological membranes [112]. ACZ-loaded TGS exhibited higher permeability than ACZ-loaded TNVs. This may be attributable to the combined effect of encapsulating ACZ in the transferomal nano-vesicles and poloxamer gel. The gel provides an occlusive effect that facilitates holding water and thus increasing the hydration level of skin for a longer period as compared with TNVs, where water evaporates at a higher rate after application of the transfersomal dispersion [113].

Table 12 shows the kinetic study of the ex vivo *corneal* permeation of the ACZ-loaded TGS, ACZ-loaded TNVs, and ACZ aqueous dispersion. The results depicted that the ex vivo permeation through the corneal membrane from different formulations followed Higuchi model kinetics according to the calculated correlation coefficient values [110].

### 3.11. In Vivo Study for ACZ ACZ-Loaded TGS

#### 3.11.1. Ocular Irritancy Test

The ocular irritancy test exhibited the absence of any signs of inflammation, such as redness, increased tear production, or edema, verifying the safety of the excipients used for topical application into the eye. Moreover, these results may be attributed to the small size of the transfersomal vesicles and the perfect encapsulation and dispersion of the drug in them [56].

#### 3.11.2. Pharmacodynamic Study

**Figure 13:**
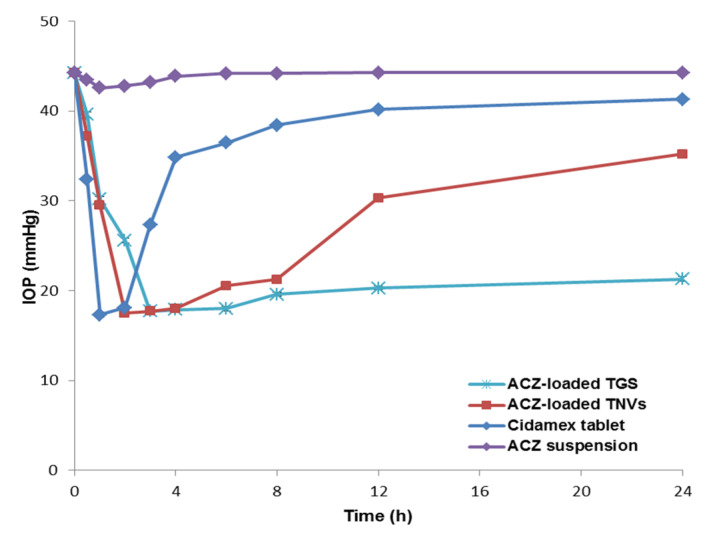
IOP-lowering effect of ACZ-loaded TGS compared to TNVs, free drug suspension, and marketed product (*n* = 3); dose of different formulations is equivalent to 500 µg of ACZ. Abbreviation: IOP, intraocular pressure; TGS, transgelosome; TNVs, transfersomal nanovesicles.

Figure 13 investigates the variation in the measured IOP over a follow-up period of 24 h. The average baseline value of the IOP for the hypertensive rabbits was 44.3 ± 3.4 mmHg. The contralateral left eye was kept at the normal level of 20.4 ± 1.9 mmHg and that demonstrated the local effect of ACZ. Weak and non-significant decrease in the IOP was detected in case of ACZ suspension. This may be attributed to poor corneal permeation of ACZ and its rapid drainage from the eyes [5]. Both the ACZ-loaded TNVs and the ACZ-loaded TGS exhibited a significant lowering in IOP compared with the free ACZ dispersion with *p* < 0.05. For the ACZ-TNVs, the IOP decreased to normal range after 2 h of administration, and it was kept within the normal levels for 6 h, and then the IOP gradually increased above the normal range. ACZ-loaded TGS produced a more significant and prolonged reduction in IOP. The IOP of ACZ-loaded TGS decreased to normal value after 3 h of administration, and the IOP reduction was sustained during the study period of 24 h. That may be attributed to improved drug permeation and increased precorneal residence time by decreasing drug drainage, leading to higher bioavailability [5].

By contrast, after the administration of Cidamex^®^ tablet, the IOP decreased immediately to normal value after 1 h of administration. However, after 3 h of administration, it increased above the normal value over the rest of the period of observation, and the IOP-lowering effect was nearly abolished.

From the above results, it is obvious that the effect of the ACZ-loaded TGS was more significant and sustained for a period of 24 h. These results revealed that TGS formulations are efficient prolonged-release drug carriers that could enhance ACZ permeation and increased its precorneal residence time, causing a more pronounced and prolonged reduction in IOP than the free drug.

#### 3.11.3. Pharmacokinetics Study in Aqueous Humor

**Figure 14:**
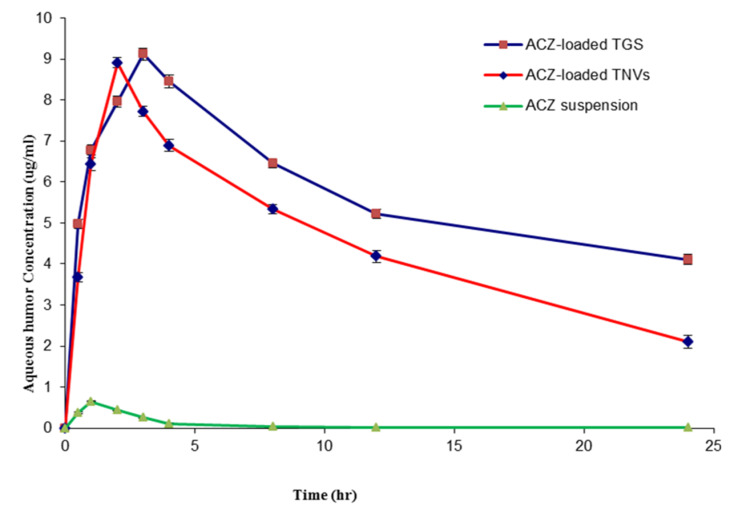
ACZ aqueous humor concentration profile after intraocular administration of different formulations (*n* = 3).

An in vivo pharmacokinetic study in the aqueous humor was performed in order to monitor the permeation properties of ACZ within the eye. Figure 14 demonstrates the concentration of ACZ in the aqueous humor after administration of different formulations.

Table 13 investigates the different pharmacokinetic parameters; the maximum aqueous humor concentration (C_max_), mean residence time (MRT), and area under the curve (AUC) of different ACZ-loaded formulations. With respect to C_max_, the results revealed that both TGS and TNVs achieved higher C_max_ compared to that of ACZ suspension (*p* < 0.001). However, no significant difference (*p* > 0.05) was detected between C_max_ of TGS and that of TNVs. The higher C_max_ of ACZ that attained post-administration of ACZ-loaded TGS or TNVs exhibited the enhancement of ACZ absorption into the aqueous humor by loading ACZ into the transfersomal vesicles.

With respect to MRT, we found that the order of increasing the MRT was as follows: ACZ-loaded TGS > TNVs > ACZ dispersion. This result exhibited the sustained release of ACZ from the gel system of TGS. Accordingly, ACZ-loaded TGS could maintain appropriate drug concentration in the aqueous humor of rabbits for 24 h that resulted in decreasing the frequency of administration and, thus, improving patient compliance. The short MRT of ACZ dispersion could be attributed on the basis of rapid drainage of ACZ from the ocular surface.

This ocular bioavailability difference was also obvious from the results of the area under the curve (AUC_0–24_) values that demonstrated a significant increase in ACZ ocular bioavailability from the two transfersomal formulations (ACZ-loaded TGS and TNVs) compared with that of the corresponding ACZ dispersion (*p* < 0.001), in addition to a further increase of the ocular bioavailability by loading the transfersomal vesicles into poloxamer gel system.

The elevated ocular bioavailability of ACZ-loaded TGS in the aqueous humor could be attributed to the synergism between the effect of encapsulating ACZ into the transfersomal vesicles and incorporation into poloxamer gel that results in increasing the permeability of ACZ in the corneal area together with the sustained release effect and prolonged contact time with the corneal area [98].

These results are in accordance with various studies that investigated the effect of nanoparticles on the pharmacokinetics of drug in aqueous humor; for instance, Ban et al. [114] found that the lipid nanoparticle of dexamethasone demonstrated higher corneal permeation and consequently enhanced ocular bioavailability compared with the aqueous solution of dexamethasone. In addition, Hassan et al. [58] reported that carvedilol-loaded leciplex formulation revealed a significant enhancement in the retention time and ocular bioavailability of carvedilol in comparison with carvedilol aqueous solution. Moreover, Xingqi et al. concluded that the liquid crystal gels of pilocarpine nitrate extended the corneal retention time and demonstrated higher ocular bioavailability than those of the drug solution.

Furthermore, El-Sayed et al. [98] reported that Flurbiprofen-loaded niosomal gel displayed higher (AUC), and thus higher ocular bioavailability than those of the corresponding niosomal dispersion and the drug solution.

## 4. Conclusions

ACZ-loaded TGS formulations were formulated to achieve better drug efficacy with minimum toxicity, by improving drug permeability and increasing the precorneal residence time and thus minimizing the adverse effects encountered with ACZ oral administration. Compared to free ACZ, the ACZ-loaded TGS prolonged drug release, improved drug permeation, increased the precorneal residence time, and achieved better drug efficacy. ACZ-loaded TGS is considered a promising platform for improving the ocular drug delivery of ACZ.

## Figures and Tables

**Table 1:** Prescreening study for formulation of ACZ-loaded TNVs, using egg yolk PC.

Formula	Lipid-to-Surfactant Ratio	Type of Surfactant	EE%
E1	90:10	Span 60	94.60 ± 1.74
E2	80:20	Span 60	90.63 ± 2.23
E3	70:30	Span 60	84.60 ± 1.25
E4	60:40	Span 60	68.50 ± 1.27
E5	90:10	Tween 80	80.04 ± 1.32
E6	80:20	Tween 80	74.32 ± 1.53
E7	70:30	Tween 80	59.12 ± 1.35
E8	60:40	Tween 80	38.33 ± 1.35
E9	90:10	Cremophor RH	88.62 ± 1.25
E10	80:20	Cremophor RH	82.17 ± 1.21
E11	70:30	Cremophor RH	73.04 ± 1.38
E12	60:40	Cremophor RH	53.18 ± 1.65
E13	90:10	Brij 35	60.32 ± 1.71
E14	80:20	Brij 35	52.24 ± 1.09
E15	70:30	Brij 35	39.11 ± 2.12
E16	60:40	Brij 35	28.61 ± 1.33

All formulations contained 1% ACZ; X1, the values are expressed as mean ±SD; *n* = 3; 90:10 ratio involves using 90 mg PC and 10 mg EA; 80:20 ratio involves using 80 mg PC and 20 mg EA; 70:30 ratio involves using 70 mg PC and 30 mg EA. Abbreviations: EE, entrapment efficiency; ACZ-loaded TNVs, Acetazolamide-loaded transfersomal nano-vesicles.

**Table 2:** Prescreening study for formulation of ACZ-loaded TNVs, using soybean PC.

Formula	Lipid-to- Surfactant Ratio	Type of Surfactant	EE%
S1	90:10	Span 60	97.30 ± 1.68
S2	80:20	Span 60	92.63 ± 2.74
S3	70:30	Span 60	88.10 ± 1.62
S4	60:40	Span 60	70.40 ± 1.16
S5	90:10	Tween 80	82.3 ± 1.64
S6	80:20	Tween 80	76.98 ± 2.72
S7	70:30	Tween 80	62.39 ± 1.55
S8	60:40	Tween 80	40.14 ± 1.14
S9	90:10	Cremophor RH	90.57 ± 2.67
S10	80:20	Cremophor RH	84.44 ± 2.82
S11	70:30	Cremophor RH	76.63 ± 0.49
S12	60:40	Cremophor RH	56.17 ± 2.13
S13	90:10	Brij 35	63.44 ± 1.52
S14	80:20	Brij 35	56.28 ± 2.11
S15	70:30	Brij 35	42.51 ± 1.45
S16	60:40	Brij 35	31.51 ± 1.45

All formulations contained 1% ACZ; X1, the values are expressed as mean ± SD; *n* = 3; 90:10 ratio involves using 90 mg PC and 10 mg EA; 80:20 ratio involves using 80 mg PC and 20 mg EA; 70:30 ratio involves using 70 mg PC and 30 mg EA. Abbreviations: EE, entrapment efficiency; ACZ-loaded TNVs, Acetazolamide-loaded transfersomal nano-vesicles.

**Table 3:** 3^2^ factorial design used for optimization of ACZ-loaded TNVs.

**Independent Variables**	**High (+1)**	**Medium (0)**	**Low (−1)**
X1	90:10	80:20	70:30
X2	Cremophor RH	Tween 80	Span 60
**Responses**	**Desirability**		
Y1	Maximize		
Y2	Maximize		

X1, Ratio of lipid to surfactant; X2, Type of surfactant; Y1, Q_8h_ (%); Y2, EE (%); 90:10 ratio involves using 90 mg PC and 10 mg EA; 80:20 ratio involves using 80 mg PC and 20 mg EA; 70:30 ratio involves using 70 mg PC and 30 mg EA. Abbreviations: ACZ-loaded TNVs, Acetazolamide-loaded transfersomal nano-vesicles.

**Table 4:** Experimental runs, independent variables, and measured responses in 3^2^ factorial design of ACZ-loaded TNVs.

Formula	Independent Variables		Dependent Variables	
	X1	X2	Y1	Y2
F1	1	−1	56.75 ± 0.82	97.30 ± 1.68
F2	0	−1	69.89 ± 1.57	92.63 ± 2.74
F3	−1	−1	85.03 ± 2.11	88.10 ± 1.62
F4	1	0	60.59 ± 1.24	82.3 ± 1.64
F5	0	0	85.48 ± 1.58	76.98 ± 2.72
F6	−1	0	91.23 ± 2.22	62.39 ± 1.55
F7	1	1	66.66 ± 0.58	90.57 ± 2.67
F8 ^#^	0	1	93.01 ± 3.76	84.44 ± 2.82
F9	−1	1	97.43 ± 1.89	76.63 ± 0.49

All formulations contained 1% ACZ; X1, ratio of lipid to surfactant; X2, type of surfactant; Y1, Q_8h_ (%); Y2, EE (%), the values are expressed as mean ± SD; *n* = 3; ^#^ Optimized Formula. Abbreviations: EE, entrapment efficiency; Q_8h_, % drug released after 8 h; ACZ-loaded TNVs, Acetazolamide-loaded transfersomal nano-vesicles.

**Table 5:** Output data of the 3^2^ factorial design of ACZ-loaded TNVs.

Responses	R^2^	Adjusted R^2^	Predicted R^2^	Adequate Precision
Q_8h_ (Y1)	0.9669	0.9470	0.8973	19.284
EE% (Y2)	0.8973	0.8357	0.6914	11.0919

Abbreviations: ACZ-loaded TNVs, Acetazolamide-loaded transfersomal nano-vesicles; R^2^, the coefficient of determination; Q_8h,_ % drug released after 8 h; EE, entrapment efficiency.

**Table 6:** ANOVA for the 3^2^ factorial design of ACZ-loaded TNVs.

Dependent Variable	Source	SS	df	Mean Square	F-Value	*p*-Value
Y1	Model	1769.45	3	589.82	48.68	0.0004
	X1	1423.11	1	1423.11	117.45	0.0001
	X2	346.34	2	173.17	14.29	0.0086
Y2	Model	791.97	3	263.99	14.56	0.0066
	X1	261.85	1	261.85	14.45	0.0126
	X2	530.12	2	265.06	14.62	0.0081

Y1: Q_8h_ (%), Y2: EE (%), X1: ratio of lipid to surfactant, X2: type of surfactant, values of Prob > F” less than 0.05 indicate that the model terms are significant. Abbreviation: ACZ-loaded TNVs, Acetazolamide-loaded transfersomal nano-vesicles; SS, sum of squares; DF, degree of freedom; MS, mean of squares.

**Table 7:** The calculated correlation coefficients for the in vitro release of ACZ-loaded TNVs employing different kinetic orders.

Formula	Zero Order	First Order	Higuchi Model	Hixson Crowell	Korsmeyer– Pappas
F1	0.9928	−0.9957	0.9967	0.9958	0.9911
F2	0.9822	−0.9874	0.9891	0.9873	0.9868
F3	0.9851	−0.9820	0.9964	0.9925	0.9904
F4	0.9746	−0.9778	0.9790	0.9781	0.9757
F5	0.9950	−0.9936	0.9993	0.9988	0.9921
F6	0.9649	−0.9679	0.9857	0.9849	0.9853
F7	0.9863	−0.9948	0.9949	0.9933	0.9944
F8	0.9620	−0.9675	0.9818	0.9789	0.9768
F9	0.9785	−0.9764	0.9945	0.9940	0.9941

Abbreviations: ACZ-loaded TNVs, Acetazolamide-loaded transfersomal nano-vesicles.

**Table 8:** Effect of storage on the properties of the optimized ACZ-loaded TNVs.

Parameter	Fresh Formula	Stored Formula
Q_8h_ (%)	93.01 ± 3.76	88.40 ± 0.89
EE (%)	84.44 ± 2.82	78.09 ± 0.96

Each value represents mean ± SD (*n* = 3), optimized ACZ-loaded TNVs is F8. Abbreviations: EE, entrapment efficiency; Q_8h,_ % drug released after 8 h; ACZ-loaded TNVs, Acetazolamide-loaded transfersomal nano-vesicles.

**Table 9:** Rheological properties of ACZ-loaded TGS.

Dilution with SLF	Maximum Viscosity (CP)	Minimum Viscosity (CP)	Thixotropic Behavior (cm^2^)	Farrow’s Constant
Before dilution with SLF	1788.57	146.34	4.2	2.21
After dilution with SLF	1784.32	143.09	4.0	2.20

Abbreviations: ACZ, Acetazolamide; TGS, transgelosomes; SLF, simulated lachrymal fluid.

**Table 10:** The calculated correlation coefficients for the in vitro release of ACZ-loaded TGS and ACZ aqueous dispersion employing different kinetic orders.

Formula	Zero Order	First Order	Higuchi Model	Hixson Crowell	Korsmeyer–Pappas
ACZ dispersion	0.9948	−0.9544	0.9924	0.9770	0.9703
ACZ-loaded TGS	0.9746	−0.9846	0.9858	0.9838	0.9841

Each value represents mean ± SD (*n* = 3).

**Table 11:** Ex vivo corneal permeation parameters of ACZ-loaded TGS, ACZ-loaded TNVs, and ACZ aqueous dispersion.

Formula	Jss (µg cm^−2^ h^−1^)	K_P_ (cm h^−1^)	ER
ACZ dispersion	3.39 ± 0.52	6.78E-05 ± 0.14	-
ACZ-loaded TNVs	15.50 ± 1.25	0.00030 ± 0.11	4.57
ACZ-loaded TGS	26.63 ± 1.75	0.00053 ± 0.17	7.86

Each value represents mean ± SD (*n* = 3). Abbreviations: J_ss_, steady state flux; K_P_, permeability coefficient; ER, enhancement ratio.

**Table 12:** The calculated correlation coefficients for the ex vivo corneal permeation of ACZ aqueous dispersion, ACZ-loaded TNVs, and ACZ-loaded TGS employing different kinetic orders.

Formula	Zero Order	First Order	Higuchi Model	Hixson Crowell	Korsmeyer–Pappas
ACZ dispersion	0.9848	−0.9863	0.9915	0.9858	0.9901
ACZ-loaded TNVs	0.9661	−0.9827	0.9962	0.9777	0.9910
ACZ-loaded TGS	0.9941	−0.9990	0.9993	0.9992	0.9900

Each value represents mean ± SD (*n* = 3).

**Table 13:** Aqueous humor pharmacokinetic parameters following ocular administration of ACZ formulations.

Parameter	ACZ Aqueous Dispersion	ACZ-Loaded TNVs	ACZ-Loaded TGS
C_max_ (µg mL^−1^)	0.651 ± 0.04	8.91 ± 0.16	9.12 ± 0.13
AUC_0–24_ (µg h mL^−1^)	1.95 ± 0.08	107.91 ± 1.32	138.08 ± 2.13
MRT (h)	3.92 ± 0.11	12.77 ± 0.45	16.76 ± 0.63

C_max_: maximum aqueous humor concentration (C_max_); AUC: area under the ACZ concentration-time curve; MRT: mean residence time.
